# Harnessing donor unrestricted T-cells for new vaccines against tuberculosis

**DOI:** 10.1016/j.vaccine.2019.04.050

**Published:** 2019-05-21

**Authors:** Simone A. Joosten, Tom H.M. Ottenhoff, David M. Lewinsohn, Daniel F. Hoft, D. Branch Moody, Chetan Seshadri, for the Collaboration for Tuberculosis Vaccine Discovery - Donor-Unrestricted T-cells Working Group, Bill and Melinda Gates Foundation

**Affiliations:** a Department of Infectious Diseases, Leiden University Medical Center, Leiden, the Netherlands; b Department of Medicine, Division of Pulmonary & Critical Care Medicine, Oregon Health Sciences University, Portland, USA; c Department of Internal Medicine, Saint Louis University, Doisy Research Center, 8th floor, 1100 S. Grand Blvd., St. Louis, MO 63104, USA; d Department of Medicine, Division of Rheumatology, Immunology and Allergy, Brigham & Women’s Hospital, Boston, Harvard Medical School, USA; e Department of Medicine, Division of Infectious Diseases, University of Washington, Seattle, USA; f Tuberculosis Research & Training Center, University of Washington, Seattle, USA

**Keywords:** Vaccine, Tuberculosis, Donor-unrestricted T-cells, Tetramers

## Abstract

*Mycobacterium bovis* bacille Calmette-Guérin (BCG) prevents extrapulmonary tuberculosis (TB) and death among infants but fails to consistently and sufficiently prevent pulmonary TB in adults. Thus, TB remains the leading infectious cause of death worldwide, and new vaccine approaches are urgently needed. T-cells are important for protective immunity to *Mycobacterium tuberculosis* (Mtb), but the optimal T-cell antigens to be included in new vaccines are not established. T-cells are often thought of as responding mainly to peptide antigens presented by polymorphic major histocompatibility complex (MHC) I and II molecules. Over the past two decades, the number of non-peptidic Mtb derived antigens for αβ and γδ T-cells has expanded rapidly, creating broader perspectives about the types of molecules that could be targeted by T-cell-based vaccines against TB. Many of these non-peptide responsive T-cell subsets in humans are activated in a manner that is unrestricted by classical MHC-dependent antigen-presenting systems, but instead require essentially nonpolymorphic presentation systems. These systems are Cluster of differentiation 1 (CD1), MHC related protein 1 (MR1), butyrophilin 3A1, as well as the nonclassical MHC class Ib family member HLA-E. Thus, the resulting T-cell responses can be shared among a genetically diverse population, creating the concept of donor-unrestricted T-cells (DURTs). Here, we review evidence that DURTs are an abundant component of the human immune system and recognize many antigens expressed by Mtb, including antigens that are expressed in BCG and other candidate whole cell vaccines. Further, DURTs exhibit functional diversity and demonstrate the ability to control microbial infection in small animal models. Finally, we outline specific knowledge gaps and research priorities that must be addressed to realize the full potential of DURTs as part of new TB vaccines approaches.

## 1 The tuberculosis vaccine challenge

*Mycobacterium tuberculosis* (Mtb) was responsible for over ten million infections and 1.6 million deaths worldwide in 2017 [1]. *M. bovis* bacille Calmette-Guérin (BCG) is the only licensed vaccine for tuberculosis (TB) and provides protection against disseminated forms of the disease in children but is inconsistent in preventing pulmonary TB in adults [2], [3]. Since adults with pulmonary TB are thought to be the primary transmitters of Mtb, control of the epidemic will require more than the current generation of BCG vaccines. A major approach to vaccination is inducing T-cell responses to Mtb antigens because several lines of evidence indicate a role for T-cell mediated immunity in controlling the clinical course of tuberculosis. Studies in mouse and non-human primate models (NHP) have demonstrated the essential role of T-cells in conferring protection against challenge with Mtb [4], [5], [6]. In natural infection, CD4^+^ T-cell depletion as a result of human immunodeficiency virus (HIV) co-infection has also been associated with increased risk of pulmonary and extrapulmonary tuberculosis [7]. However, it is not known which mycobacterial antigens are targeted by protective T-cell responses, though water soluble secreted proteins have emerged as lead candidates for subunit vaccines.

The experience to date with subunit vaccines has been mixed. The development of MVA85A, a recombinant virally vectored vaccine expressing the Mtb protein Ag85A was based on the idea that boosting T-cell immunity to a single immunodominant protein antigen would be sufficient to boost prior BCG induced protective immunity [8], [9]. However, when MVA85A was provided as a booster vaccine following priming BCG vaccination in South African infants, it failed to prevent Mtb infection and TB disease compared to BCG alone, despite the induction of polyfunctional and IL-17 producing CD4^+^ T-cells [10]. By contrast, a recombinant protein subunit vaccine known as M72 (a fusion protein composed of Mtb32A and Mtb39A) used in combination with a potent adjuvant (AS01_E_) showed over 50% protective efficacy against pulmonary TB in Mtb-infected adults [11].

Another strategy has been to use whole cell mycobacterial vaccines, such as BCG. There is demonstrated heterogeneity in the T-cell response induced by BCG, suggesting that not all antigens are recognized equally by T-cells in a genetically diverse population [12], [13]. Paradoxically, BCG vaccinated infants with a higher frequency of activated T-cells were shown to be at risk of TB disease progression [14]. Finally, revaccination of South African adolescents with BCG was shown to reduce acquisition of Mtb infection as measured by sustained conversion of an interferon-gamma release assay (IGRA) [15]. Collectively, the data suggest that while T-cells are important to controlling Mtb infection, not all vaccine strategies induce T-cells of sufficient antigenic breadth, functional diversity, or magnitude to confer protection against Mtb.

BCG is known to deliver immunogenic peptide antigens to antigen presenting cells, but the ‘whole organism’ nature of the vaccine means that non-peptide antigens are delivered as well. Generally, TB vaccinologists have not considered the potential importance of donor-unrestricted T-cells that respond to non-protein antigens produced by mycobacteria. Our goal with this review is to summarize the current state of knowledge on donor-unrestricted T cells in microbial pathogenesis, with a focus on Mtb. We outline specific knowledge gaps and research priorities which must be tackled in order to realize the full potential of non-peptide antigens in new vaccines for TB.

## 2 Donor-unrestricted T-cells (DURTs)

Peptide antigens are presented to CD8^+^ and CD4^+^ T-cells by highly polymorphic major histocompatibility complex (MHC) Class I and Class II molecules, which have been studied extensively in TB [16]. However, many T-cells can be activated by more recently discovered non-MHC antigen presenting systems. MHC related protein 1 (MR1) is a MHC Class I-like protein that presents microbial metabolites, including derivatives of vitamin B such as 5-(2-oxopropylideneamino)-6-d-ribitylaminouracil (5-OPRU) [17], [18] and certain drugs, to T-cells [17], [19], [20]. Cluster of differentiation 1 (CD1) is also structurally homologous to MHC Class I but evolved to present microbial and mammalian lipids to T-cells, including γδ T-cells [21], [22], [23], [24]. Butyrophilin 3A1 was recently shown to facilitate recognition of ‘phosphoantigens,’ small molecules like isopentenyl pyrophosphate, by γδ T-cells [25], [26]. Finally, human leukocyte antigen E (HLA-E) can present peptides and glyco-peptides from various pathogens to T-cells [27], [28], [29].

Whereas the MHC Class I and Class II genes are the most polymorphic in the human genome, MR1, CD1 and butyrophilins are virtually non-polymorphic. The known single nucleotide polymorphisms are not known to strongly affect function. Moreover, some CD1-restricted and MR1-restricted T-cells express a partially invariant T-cell receptor (TCR) that is shared across genetically unrelated individuals [30], [31]. Because the specific T-cells that recognize antigens presented by these presentation pathways are not restricted to the genome of the donor, they have acquired the moniker ‘donor-unrestricted T-cells’ or DURTs [32]. The donor-unrestricted nature of antigen presentation carries particular importance for vaccine development since a single vaccine immunogen could be designed to target the entire global population without respect to host genetic factors.

Although tools to enumerate DURTs are currently limited, estimations indicate that up to 5% of circulating peripheral human T-cells are CD1-restricted, another 5% is MR1-restricted and also an estimated 5% of peripheral T-cells are γδ T-cells [33]. In addition to the peripheral compartment, DURTs also home to tissues and may accumulate locally. MR1-restricted T-cells in particular have a phenotype that suggest preferential homing to tissues. In the liver, up to 45% of lymphocytes may be MAIT cells, but also intestines contain considerable proportions of MAIT cells [34]. γδ T-cells are abundantly present in the human skin, and represent a major T-cell population in both epidermis and dermis [35]. Intestinal intraepithelial lymphocytes are comprised of 37% γδ T-cells, thus γδ T-cells represent one of the major population of large intestinal lymphocytes [36]. Enumeration of CD1-restricted T-cells in tissue samples was limited, although autoreactive CD1-restricted T-cells do express skin homing receptors such as cutaneous lymphocyte antigen (CLA) [37]. Since HLA-E restricted T-cells recognize a large array of antigens and lack specific surface markers their absolute frequencies remain unknown [38]. However, the abundance of HLA-E recognition in experimental vaccination studies indirectly suggests that these cells may be abundantly present [39]. In the following sections, it is not our intention to broadly review DURT biology, but rather focus on specific studies that highlight their potential for TB vaccine development. Several recent reviews summarize the evolution, genetics, biochemistry, and immunology of DURTs and their ligands [33], [38], [40], [41].

## 3 Mycobacterial antigens presented by MR1

MR1 is among the most evolutionarily conserved MHC class I related molecules among mammals [42], [43]. An MR1 polymorphism resulting in low mRNA expression was associated with TB meningitis [44]. Further, mucosal associated invariant T-cells (MAIT cells) in patients with active tuberculosis show an activated phenotype, suggesting they are readily activated *in vivo*. MAIT cells recognize Mtb infected cells in an MR1-dependent manner and express a semi-invariant T-cell receptor characterized by the use of the TRAV1-2 variable region and certain joining regions [45], [46]. However, recent data suggest that MAIT cell clones expressing TCRs that are distinct from the canonical variable and joining regions can also respond to MR1-presented ligands [47]. MR1 antigens include 5-OPRU and ribityl lumazines, which are produced by many bacteria and fungi, as well as photolumazines which are produced by mycobacteria [47], [48], [49], [50].

As these antigens are small molecules derived from microbial biosynthesis, little is known at present about how best to deliver these molecules for T-cell activation *in vivo*. In particular, the most potent antigens like 5-OPRU are sensitive to degradation, so the synthesis of stable MR1 ligands is a current challenge. Two reports using mouse models have demonstrated that exogenous delivery of MR1 ligands resulted in stable expansion of lung resident MR1-restricted T-cells [51], [52]. Another study showed that challenge of human volunteers with *Salmonella paratyphi* resulted in the expansion of MR1-restricted T-cells with associated clonotypic expansions of selected MAIT-cells [53]. Whether or not these vaccine-induced expansions result in stable long-term memory populations remains to be determined.

## 4 Mycobacterial antigen presentation by CD1

Humans express four CD1 antigen presenting molecules (CD1a, CD1b, CD1c, CD1d), which vary in the configuration of their binding grooves, patterns of cellular expression, and subcellular trafficking [54]. By contrast, mice express only two nearly identical orthologs of human CD1d, so this model has only provided a narrow window into the role of CD1d-restricted T-cells in TB [4]. Evolutionary genetic analysis suggest that mice are the exception among mammals, most of which have expanded CD1 gene families similar to humans [40]. These data highlight the need to understand the distinct functions of CD1a, CD1b, and CD1c. Though CD1 proteins exhibit limited structural variation, a polymorphism in CD1a that is associated with low surface expression and T-cell activation was shown to be associated with TB [55], [56]. CD1b is expressed in TB granulomas and may locally activate lipid reactive T-cells [57].

CD1 presented antigens were first discovered in the context of mycobacteria and focused on the long chain lipid, mycolic acid, followed later by structurally related glycolipid antigens, glucose monomycolate and glycerol monomycolate, which are all presented by CD1b [23], [58], [59]. Diacylated sulfotrehalose is structurally distinct but also a mycobacterial glycolipid antigen presented by CD1b [60]. Mycobacterial lipopeptide and mycoketide antigens are presented by CD1a and CD1c, respectively [58], [61]. These lipid antigens have been chemically synthesized in high yield. Further, defining the structure of lipid ligands has facilitated studies into the molecular requirements of T-cell recognition of lipid antigens [62]. More importantly, these chemically defined reagents have enabled the development of tetramers to probe the functions of human antigen-specific T-cells, and have begun to be used as subunit vaccines in small animal models [21], [63], [64], [65], [66]. CD1b tetramers facilitated the isolation of T-cells that recognized glucose monomycolate and subsequent TCR repertoire analysis. The TCR repertoire was diverse, expanding upon previously reported conserved motifs and demonstrating clonal expansion in patients with active pulmonary TB [67]. Thousands of mycobacterial peptide antigens have been described, but the number of chemically defined CD1 lipid antigens is less than ten. Thus, much work remains to be done in comprehensively characterizing the T-cell activating lipid antigens produced by Mtb.

## 5 γδ T-cell response to Mtb

γδ T-cell subsets in humans are typically categorized according to the specific TCR-δ chain variable segment. The most abundant is the Vδ2 expressing subset, which accounts for nearly 90% of γδ T-cells in adult peripheral blood, and is usually paired with the Vγ9 gene segment [68]. Vγ9 Vδ2 T-cells are activated by alkylpyrophosphates and ‘phosphoantigens’ that are produced by mammalian cells as well as intracellular pathogens, including Mtb. Phosphoantigen recognition is mediated by butyrophylin 3A1 (BTN3A1) molecules, though the precise molecular mechanism by which this occurs has been controversial. The most recent studies suggest that phosphoantigens bind the intracellular domain of BTN3A1, leading to a conformational change on the extracellular domain, rather than being in direct contact with the T-cell receptor after binding the extracellular domain [25], [26], [69], [70].

The BTN3A1 gene expresses some degree of polymorphism, and initial sequence comparisons within the 1000 Genomes Project revealed 57 alleles encoding 33 allotypes [71]. However, most allotypes are rare, and only 8 allotypes have frequencies exceeding 1% in specific populations [71]. The IgV domain of the BTN3A1 molecule is thought to be important for the direct activation of Vγ9Vδ2 TCR, and the IgV domains expressed by BTN3A1, BTN3A2 and BTN3A3 are highly conserved and strongly homologous. These findings suggest that all 3 BTN3 molecules may be involved in regulation of Vγ9 Vδ2 T-cell activation [71]. Recently, 6-O-methylglucose-containing lipopolysaccharides (mGLP) were identified as γδ T-cell antigens specifically produced by Mtb that activate a subset of Vγ9 Vδ2 T-cells [72]. Though less abundant in blood, Vδ1 and Vδ3 expressing T-cells have been shown to recognize lipid antigens presented by CD1c and CD1d proteins [21], [22], [24], [73], [74]. Specifically, Vδ1 T-cells have been shown to recognize mycobacterial phosphomycoketide antigens presented by CD1c [24]. In addition, γδ T-cells specific for Mtb peptides have been identified that might contribute to protection [75], [76], [77]. Thus, mGLP and mycoketides could plausibly serve as specific antigens in a TB vaccine designed to activate γδ T-cells.

## 6 HLA-E presentation of Mtb antigens

HLA-E has two alleles, HLA-E*01:01 (E^R^) and *01:03 (E^G^), which differ in a single amino acid at position 107 (arginine or glycine), which is outside the peptide binding groove [78]. Whether HLA-E^R^ and HLA-E^G^ display functional differences has not been studied in detail [78], [79], but HLA-E^G^ homozygous cells express higher levels of HLA-E and had higher peptide-binding affinity. HLA-E classically presents signal sequence peptides from HLA class Ia alleles, and as such regulates innate immunity by inhibiting NK-cell activation through ligation to NKG2A/CD94 [80]. CMV peptides were the first identified ligands presented by human HLA-E and recognized in a TCR-dependent manner [81]. Subsequent work has also demonstrated that mycobacterial peptides and glycopeptides are presented by HLA-E to CD8^+^ T-cells [28], [29], [82], [83]. The crystal structure of HLA-E bound to an Mtb-derived peptide antigen revealed flexibility of the conformation of bound peptides despite preferred primary anchor residues [84]. CMV vector based TB vaccination studies in rhesus macaques suggested that there is a very high density of MHC-E (the primate equivalent of HLA-E) epitopes, with an estimated average of ∼4 epitopes per 100 amino acids, supporting the idea that additional epitopes for HLA-E presentation are yet to be discovered in the Mtb proteome [85].

## 7 Functional diversity and microbial control by DURTs

MR1-restricted T-cells have features of both innate and adaptive immune cells that preclude simple categorization. MAIT-cells in thymus and peripheral blood have the capacity to secrete proinflammatory cytokines such as IFN-γ and TNF-α, as well as cytolytic capacity [86], [87], [88]. While MAIT cells can respond to Mtb through the TCR, they can also respond to environmental signals most notably IL-12, IL-18, and TLR stimulation [47], [89], [90], [91], [92]. Thus, MR1-restricted T-cells, particularly those in close proximity to microbial infection, might possess enhanced anti-microbial functions. Notably, one study demonstrated that a murine population of CD4, CD8 double negative (DN) MAIT could inhibit mycobacterial growth in an nitric oxide dependent fashion [93]. In mice, MAIT cells have been shown to facilitate the control of *Klebsiella pneumoniae*, *Mycobacterium bovis* BCG, *Francicella tularensis*, and *Legionella longbeachiae* [52], [94], [95], [96], [97], [98]. While mice have MAIT cells, their frequency is markedly lower than seen in humans, and their phenotype seems skewed towards the production of IL-17, which is not typically seen in humans.

The initial description of the functions of CD1-restricted T-cells targeting mycobacteria were the result of studies on *in vitro* derived human T-cell clones. These showed a strong bias toward Th1 phenotypes, as characterized by the production of IFN-γ and TNF-α but not IL-4 [99]. These cells also displayed cytotoxic capacity and were able to lyse antigen-pulsed or Mtb-infected target cells [99], [100]. Studies using an *ex vivo* functional assay have confirmed and extended these findings by revealing polyfunctional phenotypes characterized by simultaneous production of IFN-γ, TNF-α, IL-2, and CD40L [101]. Total lipid reactive T-cells were detected in individuals with Mtb infection, but not in patients with active TB disease until two weeks after initiation of chemotherapy [102]. Mycolic acid specific CD1 restricted human T-cell populations producing both IFN-γ and IL-2 were detected in patients with active pulmonary TB disease, but not in BCG-vaccinated individuals without Mtb infection [103]. TCRs mediate recognition of mycobacterial phospholipids presented by CD1c, and there did not seem to be a discernable pattern even among TCRs recognizing the same or similar antigens [104]. A humanized mouse model of CD1 was recently developed that recapitulates the expression pattern and immunology of CD1a, CD1b and CD1c in humans [105]. Adoptive transfer studies in this system have shown that mycolic acid-specific T-cells can confer modest protection against Mtb challenge [106]. A guinea pig study examining aerosol Mtb challenge after vaccination with mycobacterial lipids incorporated into liposomes showed a reduction in the size but not the number of lung lesions [107]. Thus, lipid-specific T-cells induced by vaccination could plausibly provide protective immunity against Mtb in humans. However, a number of questions remain, including the functions of these T-cells *in vivo* and at sites of infection, as well as whether they can be used to generate durable immunity.

The major subsets of γδ T-cells, defined based on the γ or δ chain expressed, differ markedly between mice and humans. Therefore, it is unclear whether the literature describing γδ T cell function in mice predicts the function of human Vδ1^+^ or Vδ2^+^ T cells. In cattle, γδ T-cells accumulate quickly in the early phase of *M. bovis* infection, but decrease rapidly upon the arrival of other cells, possibly contributing to the early stages of granuloma formation [108]. Circulating γδ T-cells from *M. bovis* exposed animals produced more IFN-γ and CCL2, expressed higher amounts of cytolytic molecules, and lysed BCG-infected target cells at higher efficiency compared to naive animals [109]. Similar to the finding in humans that BCG vaccination induces expansion *in vivo* of a memory population of Vγ9 Vδ2 T-cells [110], a rapid and robust expansion of γδ T-cells was observed in BCG or Mtb-infected rhesus macaques [111], [112]. γδ T-cell responses in NHP can specifically be boosted by addition of phosphoantigens to protein subunit vaccines [113]. Adoptive transfer of γδ T-cells in NHP reduced the bacterial burden and limited disease to the infected lobe by prevention of dissemination [114]. γδ T-cells in peripheral blood of TB patients produced more IL-17 compared to those from healthy controls, however upon antigenic restimulation, more IFN-γ producing γδ T-cells were present in peripheral blood of TB patients [115]. Human γδ T-cells can reduce the burden of mycobacteria *in vitro* [116], but also provide helper functions to activate more classical components of the immune system [117]. γδ T-cells can either have direct lytic effects on mycobacteria but can also activate monocytes to produce TNF-α and thereby activate intracellular pathways to kill mycobacteria [118]. Thus γδ T-cells might be functionally important in reducing the burden of mycobacteria by direct effector activity, but may contribute indirectly by activation of other key immune players.

Human HLA-E restricted CD8^+^ T-cells express cytolytic effector molecules such as granulysin, perforin and granzymes [82], [119], and most Mtb specific HLA-E restricted CD8^+^ T-cell lines have cytolytic activity towards Mtb or BCG infected macrophages [28]. These T-cells were also able to inhibit intracellular Mtb growth in human macrophages [82]. However, these T-cells did not produce high levels of IFN-γ typical of Th-1 T-cells, but rather the hallmark Th-2 cytokines IL-4, IL-5 and IL-13 and the associated transcription factor GATA-3 [82], [119], [120]. Qa-1 is the murine homolog of HLA-E and has been shown to bind and present human HLA-E binding peptides to murine CD8^+^ T-cells with cytolytic and regulatory activity [121]. Knock-out studies confirmed a direct role for Qa-1 in mediating a protective immune response against Mtb by regulating histopathology and bacterial burden [121]. These results support and are in agreement with the above discussed human studies, and thus mice may be considered as a model system to guide HLA-E based vaccine evaluation, albeit with all general limitations of TB vaccine evaluation in mice. Presentation of Mtb peptides by HLA-E in non-human primates likely occurs because the molecules are highly conserved across primate species [122]. The first evaluation of an attenuated rhesus CMV (RhCMV) vaccine showed strong protection against simian immunodeficiency virus (SIV) infection in rhesus macaques, and this protection was due, in part, to HLA-E restricted T-cells [39], [123], [124]. Strong protection against tuberculosis was also observed following vaccination with RhCMV vectors, but HLA-E restricted T-cells were shown to be redundant in this system [85]. Together these studies suggest a contribution of HLA-E restricted T-cells to protective immunity against TB.

## 8 Harnessing DURTs for TB vaccines

For all of the DURTs discussed above, the antigenic targets, cellular presentation pathways, and molecular mechanisms of T-cell activation are now well understood. Thus, current and future research must focus on identifying the extent to which their activation results in durable immunological memory or protective effects in the acute setting. Surmounting these knowledge gaps will require a coordinated strategy by funders, academic labs, and industry (Table 1).

**Table 1:** DURT Research Priorities for TB Vaccine Development. Donor-unrestricted T-cells (DURTs) are activated by Mtb antigens presented by non-polymorphic antigen presenting systems. These include MR1, CD1, HLA-E for ɑβ T-cells and CD1 or butyrophilins for γδ T-cells. The priorities are listed roughly in the order of importance.

Antigens	Optimize chemical synthesis of non-peptide antigens to achieve high yields of pure and stable compounds
Vaccines	Formulate DURT antigens with adjuvants to achieve subunit vaccines
Tetramers	Develop and validate DURT tetramers that can be used to quantify DURT frequencies and characterize their functional and phenotypic profiles. Tetramers will be incorporated into sample sparing assays to be employed in animal and human studies as below
Animal Studies	Establish non-human primate (NHP) models to evaluate • immunogenicity and efficacy of DURT vaccines • DURT correlates of protection in Mtb challenge studies.
Human Studies	Use tetramers to evaluate DURTs as • Immune correlates of risk in natural history studies • Immune correlates of protection in vaccine efficacy studies

The first major hurdle is to ensure the availability of sufficient quantity of stable DURT antigens with which to study and optimize vaccine formulation. As described above, peptides and lipids presented by HLA-E and CD1, respectively, are already available as stable reagents in high yield. Many Mtb lipid antigens have been synthesized for the CD1 system and have been successfully validated in vitro or with tetramers [21], [64], [125], [126], [127]. However, MR1 ligands are inherently unstable and the γδ T cell ligand mGLP is complex and has yet to be synthesized.

Once pure ligands are available, the next challenge will be to determine how to formulate them as vaccines. The biochemistry governing solubility of peptides, lipids, and small molecules may preclude co-formulation and require parallel development strategies. At the same time, selection of adjuvants to include or whether adjuvants are required at all will be another challenge. Some mycobacterial lipid antigens, such as phosphotidyl-*myo*-inositol mannosides, may fortuitously act as both antigen and adjuvant by activating Toll-like receptor pathways [128], [129]. Lipid antigens may also lend themselves easily to co-formulation with lipophilic adjuvants already in clinical use, such as monophosphoryl lipid A or trehalose dibehenate [130], [131]. Nanocarriers may also be employed to enhance delivery of lipid components such as mycolic acid to phagocytic immune cells, in particular following nasal immunization [132]. Alternatively liposomes may be employed as packaging for hydrophobic molecules such as lipoarabinomannan to promote uptake and presentation by antigen presenting cells [133]. Solving challenges in antigen production, purification and delivery will require expertise in medicinal chemistry, adjuvants, and product development that is under-represented within the DURT field.

Another hurdle is to develop animal models which more faithfully represent human biology. Because of the substantial differences between humans and mice for most DURTs, as discussed above, the optimal strategy may be to focus on non-human primates (NHP). NHP faithfully reproduce the spectrum of human tuberculosis, including active pulmonary disease characterized by multicellular granulomas [134]. NHP have also been used extensively in the pre-clinical evaluation of tuberculosis vaccines [135], [136]. MAIT cell frequency and phenotype in NHP is much like that seen in humans [137]. Vγ9 Vδ2 T-cells were present in NHP and expanded after BCG vaccination [111]. CD1 studies in NHP have been limited but have suggested that a vaccine containing a liposomal formulation of a mycobacterial glycolipid antigen was immunogenic [138]. Finally, HLA-E was recently shown to be functionally conserved between NHP and humans [122]. These studies have demonstrated proof-of-concept for all four DURT populations, and now a coordinated effort is required to develop and validate the reagents required to study DURTs in NHP. This may take the form of human tetramers that have cross-species reactivity or species-specific tetramers, as was demonstrated for MR1 [137]. Tetramers would facilitate immunogenicity studies of vaccine formulations described above or investigation of immunodominance among DURTs after whole cell vaccination. We could also evaluate DURT vaccines as a ‘booster’ after BCG, which would be the most likely implementation in TB endemic countries. As with humans, a major advantage of DURT tetramers in NHP will be their application across a genetically diverse population.

## 9 Human studies with whole cell mycobacterial vaccines

Despite these challenges, we have an immediate opportunity to study DURTs as correlates of protective immunity. The clinical efficacy signals observed in the recently published trials using BCG revaccination and M72 provide an opportunity to study the association of DURTs with protection from Mtb infection and pulmonary TB, respectively [11], [15]. These studies could be modeled on immune correlate studies of the RV144 vaccine for HIV, which compared the results of several standardized and validated assays [139]. Even though tetramers are available for CD1, MR1, and HLA-E, very few of these have been incorporated into assays that have undergone the rigorous process of standardization required for inclusion as an endpoint assay in a clinical trial [63], [139]. This expertise is not typically present within academic labs practicing discovery science, so may require partnerships with assay developers in industry. Tetramer-based assays would find utility in a number of ongoing clinical trials evaluating whole cell vaccines which are poly-antigenic and would be expected to boost DURTs. SRL-172 is a heat killed non-tuberculous mycobacterial vaccine that was shown to prevent tuberculosis in a large study of HIV-infected adults in Tanzania [140]. A reformulated version known as DAR-901 has completed Phase I studies and is now in Phase II [141]. VPM1002 is a recombinant *M. bovis* BCG strain that has been engineered to express bacterial listeriolysin and showed enhanced immunogenicity in a Phase I study and is currently in Phase II/III evaluation [142]. Finally, MTBVAC is an attenuated strain of Mtb that is currently in Phase II trials [143].

As an extension of these immune correlate studies, which would necessarily be performed retrospectively, DURTs might be evaluated in studies of controlled human mycobacterial challenge. These studies have already begun using live BCG [144], [145]. A whole cell vaccine for malaria consisting of irradiated sporozoites was shown to be highly effective at preventing blood stage malaria in controlled human malaria infection [146]. In follow up studies, γδ T-cells were shown to be associated with vaccine induced protection [147], [148]. Specifically, the frequency of Vδ2 expressing subset of γδ T-cells before vaccination was correlated with decreased parasitemia after immediate (3 weeks) and late (21–25 weeks) challenge with *Plasmodium falciparum* [147]. As described above, Vδ2^+^ T-cells have also been shown to be important in controlling Mtb infection in vitro, in particular in the early stages post infection, likely bridging innate and adaptive (αβ T-cells) [116], [118], [147]. A broader study of DURTs in a human TB challenge model would require validated reagents and assays, as described above.

## 10 Summary

In summary, targeting DURTs offers a complementary path to traditional strategies for inducing classical T-cell immunity to peptide antigens by increasing the breadth of targeted antigens, the functions of induced T-cells and exploiting the essential monomorphism of the antigen presentation systems involved. Whole cell mycobacterial vaccines that naturally contain DURT antigens may mediate their protective effect through the induction of DURTs. On the other hand, DURT antigens themselves may be of direct interest as vaccine immunogens. Coordination among funders, academic labs, and industry partners with expertise in product development will be required to overcome the hurdles in chemistry, animal models, and assay optimization outlined above to fully realize the potential of DURTs for new TB vaccines.

## Declaration of interests

The authors declare that they have no known competing financial interests or personal relationships that could have appeared to influence the work reported in this paper.
